# Low-dose high-resolution chest CT in adults with cystic fibrosis: intraindividual comparison between photon-counting and energy-integrating detector CT

**DOI:** 10.1186/s41747-024-00502-9

**Published:** 2024-09-19

**Authors:** Marko Frings, Matthias Welsner, Christin Mousa, Sebastian Zensen, Luca Salhöfer, Mathias Meetschen, Nikolas Beck, Denise Bos, Dirk Westhölter, Johannes Wienker, Christian Taube, Lale Umutlu, Benedikt M. Schaarschmidt, Michael Forsting, Johannes Haubold, Sivagurunathan Sutharsan, Marcel Opitz

**Affiliations:** 1 Institute of Diagnostic and Interventional Radiology and Neuroradiology, University Hospital Essen, Essen, Germany; 2 Department of Pulmonary Medicine, University Hospital Essen-Ruhrlandklinik, Essen, Germany; 3 Adult Cystic Fibrosis Center, Department of Pulmonary Medicine, University Hospital Essen-Ruhrlandklinik, Essen, Germany

**Keywords:** Cystic fibrosis, Radiation dosage, Radiation exposure, Technology (radiologic), Tomography (x-ray computed)

## Abstract

**Background:**

Regular disease monitoring with low-dose high-resolution (LD-HR) computed tomography (CT) scans is necessary for the clinical management of people with cystic fibrosis (pwCF). The aim of this study was to compare the image quality and radiation dose of LD-HR protocols between photon-counting CT (PCCT) and energy-integrating detector system CT (EID-CT) in pwCF.

**Methods:**

This retrospective study included 23 pwCF undergoing LD-HR chest CT with PCCT who had previously undergone LD-HR chest CT with EID-CT. An intraindividual comparison of radiation dose and image quality was conducted. The study measured the dose-length product, volumetric CT dose index, effective dose and signal-to-noise ratio (SNR). Three blinded radiologists assessed the overall image quality, image sharpness, and image noise using a 5-point Likert scale ranging from 1 (deficient) to 5 (very good) for image quality and image sharpness and from 1 (very high) to 5 (very low) for image noise.

**Results:**

PCCT used approximately 42% less radiation dose than EID-CT (median effective dose 0.54 *versus* 0.93 mSv, *p* < 0.001). PCCT was consistently rated higher than EID-CT for overall image quality and image sharpness. Additionally, image noise was lower with PCCT compared to EID-CT. The average SNR of the lung parenchyma was lower with PCCT compared to EID-CT (*p* < 0.001).

**Conclusion:**

In pwCF, LD-HR chest CT protocols using PCCT scans provided significantly better image quality and reduced radiation exposure compared to EID-CT.

**Relevance statement:**

In pwCF, regular follow-up could be performed through photon-counting CT instead of EID-CT, with substantial advantages in terms of both lower radiation exposure and increased image quality.

**Key Points:**

- Photon-counting CT (PCCT) and energy-integrating detector system CT (EID-CT) were compared in 23 people with cystic fibrosis (pwCF).
- Image quality was rated higher for PCCT than for EID-CT.
- PCCT used approximately 42% less radiation dose and offered superior image quality than EID-CT.

**Graphical Abstract:**

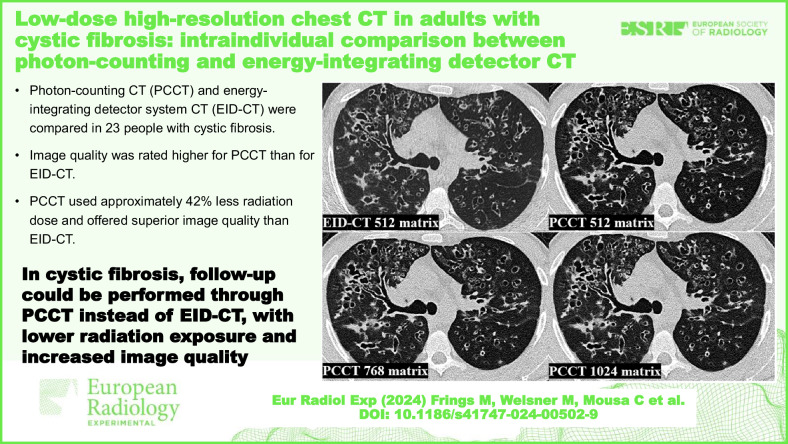

## Background

Cystic fibrosis (CF) is a hereditary condition caused by autosomal recessive mutations in the cystic fibrosis transmembrane conductance regulator (CFTR) gene. The CFTR protein encodes an ion channel for chloride and bicarbonate located on the apical surface of secretory epithelia [1–3]. The mutation causes ion shifts that increase the viscosity of extracellular mucus [1, 4–6]. Manifestations of CF include pancreatic insufficiency with gastrointestinal malabsorption and diabetes, chronic sinusitis, hepatobiliary disease, male infertility, high chloride concentration in sweat, and obstructive lung disease with bronchiectasis and repetitive bacterial infections [6, 7]. Progressive lung disease is the major cause of morbidity and mortality in people with cystic fibrosis. Chest CT examinations help CF caregivers detect the presence and progression of structural lung abnormalities at an early stage [8, 9]. CT scans are used to assess bronchiectasis with bronchial wall thickening, mucus impaction, infiltrates, air trapping and emphysema in order to guide treatment and prevent further disease progression. Disadvantageous is the method-related radiation exposure [10]. Improved treatment options, such as CFTR modulator therapy with Elexacaftor/Tezacaftor/Ivacaftor, have increased the life expectancy of people with cystic fibrosis (pwCF), and the cumulative radiation dose from lifelong CT scans for disease monitoring has become relevant in terms of malignancy risk [8, 11–13].

Numerous research studies have highlighted the significance of low-dose (LD) CT scan protocols in reducing radiation exposure to patients while maintaining diagnostic image quality [14–16]. In this context, photon-counting CT (PCCT) represents a further development in terms of radiation reduction and spatial resolution. Conventional energy-integrating detectors (EID) use scintillator detectors to convert the incoming photon into a light signal, which is then transduced into an electrical signal at a photodiode. PCCT uses a cadmium electrode as a semiconductor to transduce the energy of the photon directly into an electrical signal [17, 18]. PCCT is now in routine clinical use, and initial studies have already demonstrated a dose reduction with at least equivalent image quality [19–22].

To our knowledge, our study is the first to examine LD chest CT at PCCT in adult pwCF. The aim of this study is to investigate the radiation dose and image quality of PCCT compared to EID-CT in adult pwCF [23].

## Methods

### Study population and ethics statement

In this retrospective single-centre study, 23 pwCF underwent low-dose high-resolution (LD-HR) chest CT scans using PCCT (NAEOTOM Alpha, Siemens Healthineers, Erlangen, Germany) as part of clinical routine care between July and November 2023. All of them had previously received LD chest CT scans without high-resolution reconstruction, utilising a standard EID-CT scanner (SOMATOM Definition AS 64, Siemens Healthineers, Erlangen, Germany), which was used for comparison.

The study was conducted in accordance with the guidelines of the Declaration of Helsinki and received approval from the institutional review board (23-11602-BO). Informed consent was waived due to the retrospective study design. All chest CT scans were conducted using standard clinical protocols for diagnostic purposes, and patient information was anonymised.

### CT protocols and image acquisition

All pwCF underwent LD-HR chest CT scan using PCCT. The PCCT scan parameters were set as follows: tube voltage 100 kVp with tin filter, detector configuration 144 × 0.4 mm, automatic tube current modulation (CARE, Siemens Healthineers, Erlangen, Germany) and spiral pitch factor 1.5. For SOMATOM Definition AS 64, the following scan parameters were used: tube voltage 120 kVp with automatic tube current modulation (CARE Dose4D, Siemens Healthineers, Erlangen, Germany), detector configuration 64 mm × 0.6 mm, and spiral pitch factor 0.6.

Images were reconstructed for lung tissue using a BI64 convolution kernel (window settings centre: -350 and width: 1,500) with a slice thickness of 1 mm in axial slices for both scanners. The PCCT scans were reconstructed in a 512 × 512, 768 × 768 and 1,024 × 1,024 matrix (hereafter referred to as 512 matrix, 768 matrix and 1,024 matrix), whereas the SOMATOM Definition AS 64 images were only reconstructed in a 512 matrix due to technical limitations. The images of the PCCT were reconstructed using quantum iterative reconstruction (level 3). The images of the SOMATOM Definition AS 64 were reconstructed without iterative reconstruction. Based on the patient’s size in the scout view, the technologist manually adjusted the field of view.

### Quantitative image quality analysis

Regions of interest (ROIs) were manually placed in the same location for each patient in the healthy lung parenchyma, the autochthonous back muscles and in the air outside the patient, where materials remained consistent between pairs of CT studies. The measurement was conducted on a single slice. The signal from the ROI was measured in Hounsfield units (HU). Image noise was defined as the standard deviation of the ROI. The signal-to-noise ratio (SNR) was calculated as the quotient of the average HU in the ROI and the corresponding standard deviation for each ROI. In the analytical evaluation, the images of the EID-CT were first compared with the images of the PCCT with a 512 matrix. Then, the images with 768 and 1,024 matrix were compared with the images of the 512 matrix on the PCCT. Subsequently, the images with a 768 and 1,024 matrix were compared with each other.

### Qualitative image quality analysis

Three blinded radiologists independently evaluated the overall image quality, image sharpness and image noise of CT chest scans of 23 pwCF using a 5-point Likert scale. The radiologists had 4 years (M.F.), 5 years (H.T.), and 6 years (M.O.) of experience in chest CT. For image quality and sharpness, the scale was defined as: 1—insufficient, 2—sufficient, 3—satisfactory, 4—good, 5—very good. For image noise, the scale was: 1—very high, 2—high, 3—moderate, 4—low, 5—very low. A total of 92 reconstructions (four reconstructions per patient using PCCT BI64 convolution kernel with a slice thickness of 1 mm in a 512, 768 and 1,024 matrix and EID-CT BI64 convolution kernel with a slice thickness of 1 mm in 512 matrix) were assessed. The CT scans were anonymised and blinded before presentation to the readers. Reconstructions were presented to the three readers in random order to prevent the comparison of reconstructions within a given patient case.

### Radiation dose

To assess radiation dose, we used an automated dose monitoring software (Radimetrics Enterprise Platform, Bayer Healthcare, Leverkusen, Germany) based on Monte Carlo simulation techniques. The software retrieves radiation exposure metadata and patient demographic information from the Digital Imaging and Communications in Medicine (DICOM) header stored in the picture archiving and communication system (PACS) [24]. Effective dose was assessed using the volumetric CT dose index (CTDIvol), scan length and dose length product (DLP). To determine cancer risk for specific organs, organ doses were determined for the heart, lungs, skin, thyroid gland and red bone marrow. The dose monitoring software employed Monte Carlo simulation to calculate effective radiation doses and organ doses. The calculation involved the application of the weighting factors specified in the International Commission on Radiological Protection (ICRP) publication 103 [25, 26].

### Statistical analysis

Statistical analysis was conducted using RStudio (Version 2023.12.0). Normally distributed data are presented as mean ± standard deviation and non-normally distributed data as median and interquartile range. To assess normal distribution, the Kolmogorov–Smirnov test was used. A paired two-sided *t*-test was employed for normally distributed data and a Wilcoxon test for non-normally distributed data. Fleiss’ Kappa was computed to measure inter-rater reliability (IRR) with interpretation as follows: < 0.00 poor agreement, 0.00–0.20 slight agreement, 0.21–0.40 fair agreement, 0.41–0.60 moderate agreement, 0.61–0.80 substantial agreement, and > 0.81 almost perfect agreement [27]. Statistical significance was defined as *p*-value < 0.05 for single tests, and for multiple comparisons, the Bonferroni method was applied.

## Results

### Study population

The cohort comprised 4 women (17%) and 19 men (83%) with a mean age of 38.1 ± 12.2 years (range: 26–75 years, Table 1). The mean interval between the two CT scans (PCCT and EID-CT) was 2.49 ± 1.25 years, with a maximum span of 5.10 years and a minimum span of 0.74 years. There was no statistically significant difference in BMI between the two study time points (BMI PCCT: 21.9 ± 2.8, BMI EID-CT: 21.5 ± 2.3, *p* = 0.631, Table 1).

**Table 1:** Patient characteristics

Parameter	Value
Number	23
Age, years, mean ± SD (range)	38.1 ± 12.1 (26–75)
Female sex, n (%)	4 (17)
Genotype, number (%)	
F508del homozygous	8 (35)
F508del heterozygous	4 (17)
Other	11 (48)
BMI of patients undergoing PCCT, mean ± SD	21.9 ± 2.8*
BMI of patients undergoing EID-CT, mean ± SD	21.5 ± 2.3*

*EID-CT* Energy-integrating detector Computed tomography, *PCCT* Photon-counting computed tomography, *SD* Standard deviation

* *p*-value = 0.631

### Quantitative image quality analysis

**Fig. 1:**
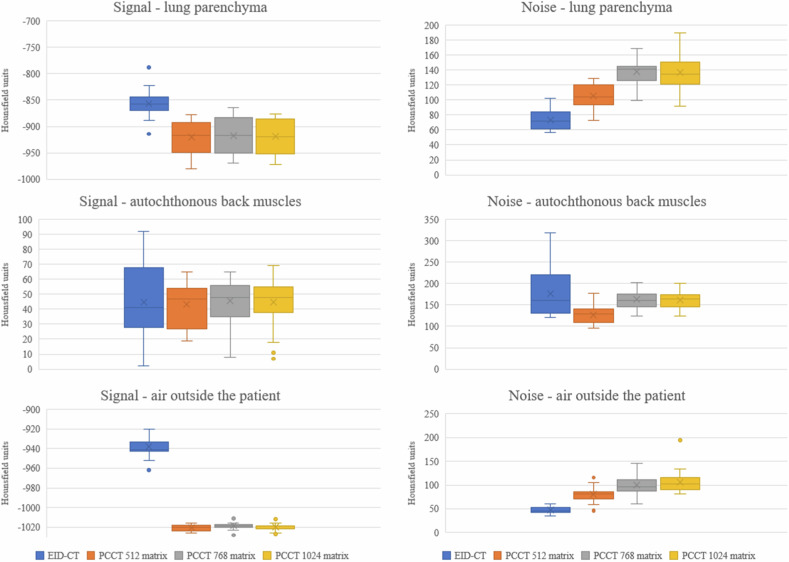
Comparison of image signal and image noise. EID-CT, Energy-integrating detector CT; PCCT, Photon-counting CT; PCCT 512, PCCT 512 × 512 matrix; PCCT 768, PCCT 768 × 768 matrix; PCCT 1,024, PCCT 1,024 × 1,024 matrix

The signal for the lung is stronger on the PCCT than on the EID-CT, whereas the noise increases continuously from the EID-CT to the 1,024 matrix on the PCCT (Fig. 1). Therefore, the EID-CT images exhibited the highest SNR of the lung parenchyma with a mean ± standard deviation of -11.99 ± 2.12, which was significantly higher than the SNR of the 512 matrix on PCCT images (PCCT 512 matrix: -8.93 ± 1.43; *p* < 0.001, Table 2). Additionally, the SNR of the lung parenchyma in PCCT with 512 matrix (-8.93 ± 1.43) was significantly higher than that in PCCT images with 768 and 1,024 matrices (PCCT 768 matrix: -6.78 ± 0.99; *p* < 0.001 and PCCT 1,024 matrix: -6.87 ± 1.12; *p* < 0.001, Table 2). There was no statistically significant difference in SNR between the 768 and 1,024 matrices (*p* = 0.681, Table 2).

**Table 2:** Quantitative image quality analysis

	ROI EID-CT 512 matrix (HU)	ROI PCCT 512 matrix (HU)	ROI PCCT 768 matrix (HU)	ROI PCCT 1,024 matrix (HU)
Lung	-857.04 ± 73.48	-920.48 ± 105.48	-916.91 ± 137.74	-919.39 ± 137.13
Autochthonous back muscles	44.87 ± 176.91	43.43 ± 126.22	45.70 ± 167.78	44.96 ± 162.04
Air outside the patient	-938.30 ± 46.96	-1,020.61 ± 79.87	-1,018.57 ± 99.65	-1,019.74 ± 106.70

	SNR EID-CT 512 matrix	SNR PCCT 512 matrix	SNR PCCT 768 matrix	SNR PCCT 1,024 matrix
Lung	-11.99 ± 2.12	-8.93 ± 1.43	-6.78 ± 0.99	-6.87 ± 1.12
Autochthonous back muscles	0.27 ± 0.17	0.35 ± 0.14	0.29 ± 0.09	0.28 ± 0.11
Air outside the patient	-20.39 ± 3.06	-13.27 ± 2.85	-10.60 ± 2.13	-9.93 ± 1.77
Statistical comparison of SNR of EID-CT and PCCT				
Lung SNR	EID-CT 512 matrix *versus* PCCT 512 matrix	PCCT 512 matrix *versus* PCCT 768 matrix	PCCT 512 matrix *versus* PCCT 1,024 matrix	PCCT 768 matrix *versus* PCCT 1,024 matrix
*p*-value	< 0.001	< 0.001	< 0.001	0.681
Autochthonous back muscles SNR	EID-CT 512 matrix *versus* PCCT 512 matrix	PCCT 512 matrix *versus* PCCT 768 matrix	PCCT 512 matrix *versus* PCCT 1,024 matrix	PCCT 768 matrix *versus* PCCT 1,024 matrix
*p*-value	0.975	0.007	0.001	0.692
Air outside the Patient SNR	EID-CT 512 matrix *versus* PCCT 512 matrix	PCCT 512 matrix *versus* PCCT 768 matrix	PCCT 512 matrix *versus* PCCT 1,024 matrix	PCCT 768 matrix *versus* PCCT 1,024 matrix
*p*-value	< 0.001	< 0.001	< 0.001	0.056

Values are mean ± standard deviation (signal ± noise)

*EID-CT* Energy-integrating detector CT, *EID-CT 512* EID-CT 512 × 512 matrix, *PCCT* Photon-counting CT, *PCCT 512* PCCT 512 × 512 matrix, *PCCT 768* PCCT 768 × 768 matrix, *PCCT 1,024* PCCT 1,024 × 1,024 matrix, *SNR* Signal-to-noise ratio

The signal strength of the autochthonous back muscles appears consistent between PCCT and EID-CT scans. The noise is lowest at the PCCT with 512 matrix and then increases to approximately the same level with the 768 matrix and 1,024 matrix and reaches the highest level on EID-CT (Fig. 1). The SNR of the autochthonous back was lower on the EID-CT images, with mean ± standard deviation of 0.27 ± 0.17 compared to PCCT images with a 512 matrix without statistical significance (0.35 ± 0.14, *p* = 0.975, Table 2). The images with a 512 matrix on PCCT had a statistically significant higher SNR compared to 768 and 1,024 matrices on PCCT (768 matrix: 0.29 ± 0.09, *p* = 0.007, PCCT 1,024 matrix: 0.28 ± 0.11, *p* = 0.001, Table 2). There was no statistical significance on the SNR between 768 and 1,024 matrices (*p* = 0.692, Table 2).

The signal strength for the air outside the patient appears higher on PCCT compared to EID-CT, while the noise level consistently rises from EID-CT to the 1,024 matrix setting on PCCT (Fig. 1). The SNR of air outside the patient was significantly lower on PCCT with a 512 matrix (-13.27 ± 2.85) than on EID-CT (-20.39 ± 3.06, *p* < 0.001, Table 2). The SNR of the 768 and 1,024 matrices was significantly lower than that of the 512 matrix (*p* < 0.001, Table 2). There was no significant difference in the SNR between the 768 and 1,024 matrices (*p* = 0.056, Table 2).

### Qualitative image quality analysis

**Fig. 2:**
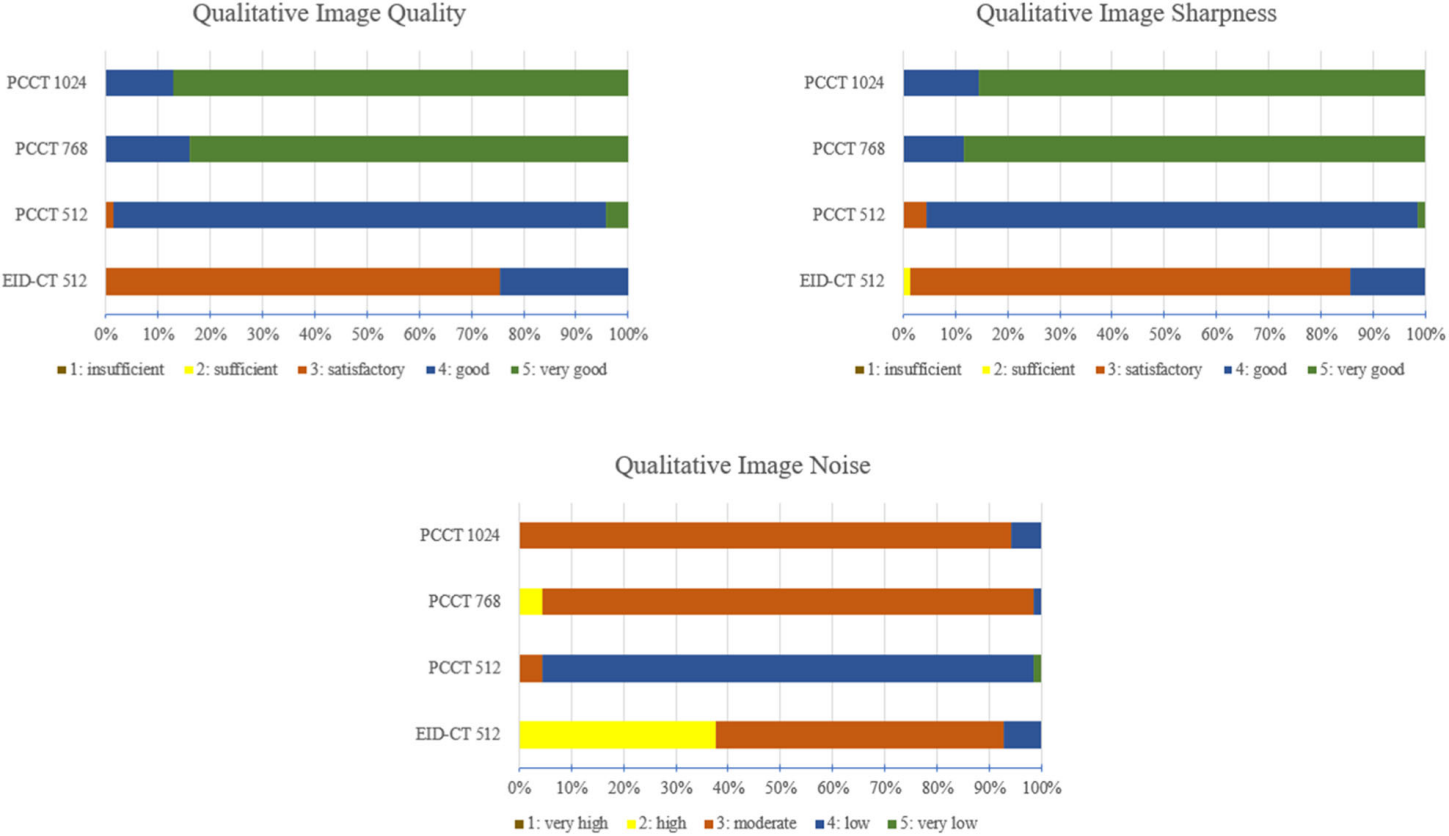
Ratings of qualitative image quality analysis. EID-CT, Energy-integrating detector CT; EID-CT 512, EID-CT 512 × 512 matrix; PCCT, Photon-counting CT; PCCT 512, PCCT 512 × 512 matrix; PCCT 768 PCCT 768 × 768 matrix; PCCT 1,024, PCCT 1,024 × 1,024 matrix

The image quality of the whole lung was rated better among PCCT images than on the EID-CT images (Fig. 2, PCCT 512 matrix, PCCT 768 matrix and PCCT 1,024 matrix: each *p* < 0.001, Table 3). For PCCT, the 768 and 1,024 matrix images were better evaluated than the 512 matrix images (Fig. 2, PCCT 768 matrix and PCCT 1,024 matrix: *p* < 0.001, Table 3). There was no significant difference in the image quality rating between the 768 and 1,024 matrix (*p* = 0.265, Table 3). The IRR demonstrated substantial agreement across all reconstructions (Table 3).

**Table 3:** Qualitative image quality analysis with statistical comparison of EID-CT and PCCT

	EID-CT 512 matrix	PCCT 512 matrix	PCCT 768 matrix	PCCT 1,024 matrix	
Image quality	3 [0]	4 [0]	5 [0]	5 [0]	
Image sharpness	3 [0]	4 [0]	5 [0]	5 [0]	
Image noise	3 [1]	4 [0]	3 [0]	3 [0]	
Image quality	EID-CT 512 matrix	PCCT 512 matrix	PCCT 768 matrix	PCCT 1,024 matrix	IRR
EID-CT 512 matrix	1	< 0.001	< 0.001	< 0.001	0.716
PCCT 512 matrix	< 0.001	1	< 0.001	< 0.001	0.737
PCCT 768 matrix	< 0.001	< 0.001	1	0.265	0.784
PCCT 1,024 matrix	< 0.001	< 0.001	0.265	1	0.744
Image sharpness	EID-CT 512 matrix	PCCT 512 matrix	PCCT 768 matrix	PCCT 1,024 matrix	IRR
EID-CT 512 matrix	1	< 0.001	< 0.001	< 0.001	0.843
PCCT 512 matrix	< 0.001	1	< 0.001	< 0.001	0.737
PCCT 768 matrix	< 0.001	< 0.001	1	0.755	0.859
PCCT 1,024 matrix	< 0.001	< 0.001	0.755	1	0.735
Image noise	EID-CT 512 matrix	PCCT 512 matrix	PCCT 768 matrix	PCCT 1,024 matrix	IRR
EID-CT 512 matrix	1	< 0.001	0.007	< 0.001	0.881
PCCT 512 matrix	< 0.001	1	< 0.001	< 0.001	0.738
PCCT 768 matrix	0.007	< 0.001	1	0.041	0.738
PCCT 1,024 matrix	< 0.001	< 0.001	0.041	1	0.735

Values are median [interquartile range], Bonferroni corrected *p*-value < 0.005 (10 comparisons)

*EID-CT* Energy-integrating detector CT, *EID-CT 512* EID-CT 512 × 512 matrix, *IRR* Inter-rater reliability, *PCCT* Photon-counting CT, *PCCT 512* PCCT 512 × 512 matrix, *PCCT 768* PCCT 768 × 768 matrix, *PCCT 1,024* PCCT 1,024 × 1,024 matrix

The image sharpness of the whole lung was rated better on the PCCT images than on the EID-CT images (Fig. 2, PCCT 512 matrix, PCCT 768 matrix and PCCT 1,024 matrix: each *p* < 0.001, Table 3). For PCCT, the 768 and 1,024 matrix images were evaluated as sharper than 512 matrix images (Fig. 2, PCCT 768 matrix and PCCT 1,024 matrix: *p* < 0.001, Table 3). There was no significant difference in the image sharpness ratings between the 768 and 1,024 matrices (*p* = 0.755, Table 3). The IRR showed at least a substantial agreement for all reconstructions (Table 3).

Image noise was rated lowest for the PCCT images with a 512 matrix, followed by the 768 and 1,024 matrices (Fig. 2). The 512 matrix of the EID-CT had the highest image noise. In summary, all PCCT reconstructions were rated with significantly lower noise than the EID-CT reconstruction (PCCT 512 matrix: *p* < 0.001, PCCT 768 matrix: *p* = 0.007 and PCCT 1,024 matrix: *p* < 0.001, Table 3). The IRR exhibited at least a substantial agreement for all reconstructions (Table 3).

**Fig. 3:**
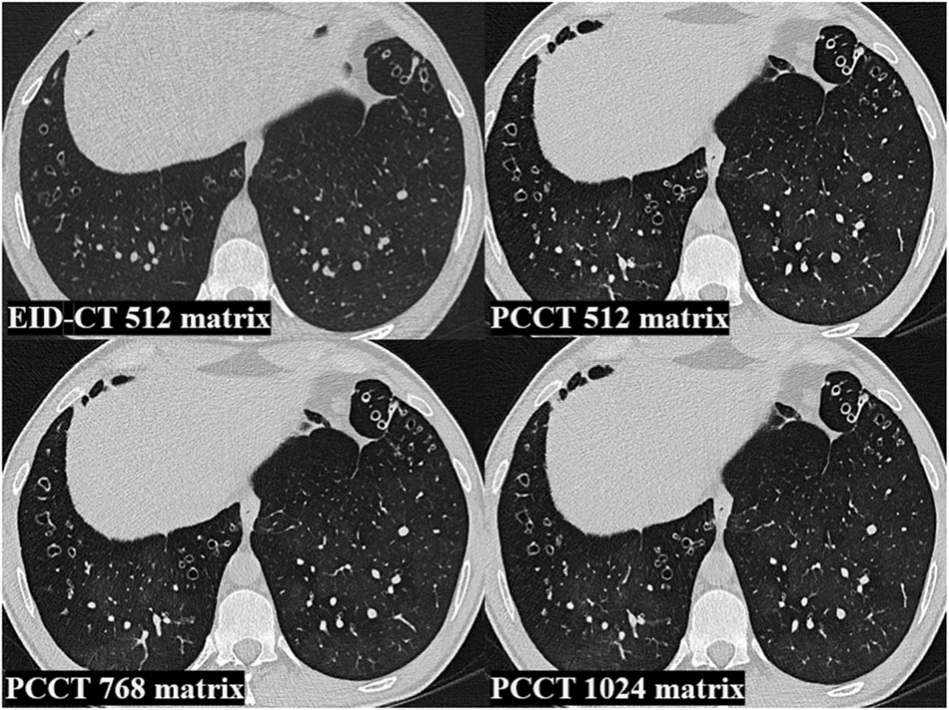
Examples of different reconstructions of chest CT of lower lobes with mosaic pattern. EID-CT, Energy-integrating detector CT; EID-CT 512, EID-CT 512 × 512 matrix; PCCT, Photon-counting CT; PCCT 512, PCCT 512 × 512 matrix; PCCT 768, PCCT 768 × 768 matrix; PCCT 1,024, PCCT 1,024 × 1,024 matrix

**Fig. 4:**
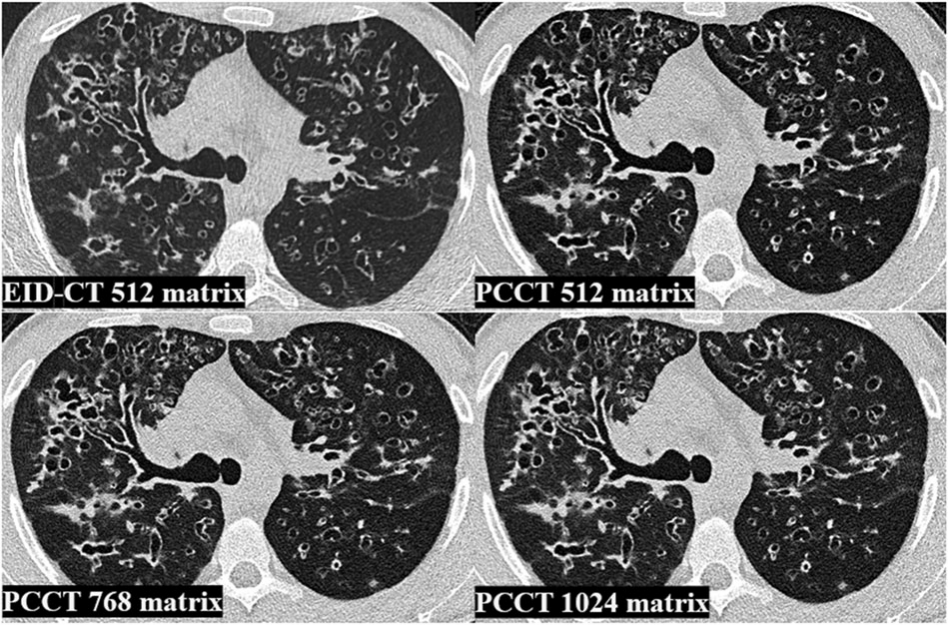
Examples of different reconstructions of chest CT of lower lobes with bronchiectasis and bronchial wall thickening. EID-CT, Energy-integrating detector CT; EID-CT 512; EID-CT 512 × 512 matrix; PCCT, Photon-counting CT; PCCT 512, PCCT 512 × 512 matrix; PCCT 768, PCCT 768 × 768 matrix; PCCT 1,024, PCCT 1,024 × 1,024 matrix

Figures 3 and 4 represent examples of different reconstructions with different findings on chest CT. It is worth noting that, in addition to the improved image quality and sharpness, a mosaic pattern is easier to detect on 768 and 1,024 matrices PCCT than on EID-CT and 512 matrix PCCT.

### Radiation dose

The CTDIvol of PCCT scans was significantly lower than that of EID-CT scans (PCCT: 0.82 [0.18] mGy, EID-CT: 1.38 [0.52] mGy, *p* < 0.001, Table 4). Similarly, the DLP of PCCT scans was significantly lower than that of EID-CT scans (PCCT: 31.60 [5.65] mGy·cm, EID-CT 48.50 [16.26] mGy·cm, *p* < 0.001, Table 4). Furthermore, the effective dose of PCCT scans was significantly lower than that of EID-CT scans (PCCT: 0.54 [0.06] mSv, EID-CT: 0.93 [0.24] mSv, *p* < 0.001, Table 4). This represents a decrease of 42%. Effective organ doses were also lower with PCCT than with EID-CT. For example, the effective dose to the lung was significantly lower with PCCT (1.23 [0.14] mSv) than with EID-CT (2.07 [0.72] mSv, *p* < 0.001, Table 4). Similarly, the effective dose to the red bone marrow was significantly lower with PCCT (0.42 [0.06] mSv) than with EID-CT (0.65 [0.21] mSv, *p* < 0.001, Table 4).

**Table 4:** Radiation dose

	EID-CT	PCCT	Difference	Percentage difference	*p*-value
CTDIvol (mGy)	1.38 [0.52]	0.82 [0.18]	0.56	0.41	< 0.001
DLP (mGy × cm)	48.50 [16.26]	31.60 [5.65]	16.90	0.35	< 0.001
Effective dose (mSv)	0.93 [0.24]	0.54 [0.06]	0.39	0.42	< 0.001
Effective organ dose					
Heart (mSv)	2.04 [0.68]	1.16 [0.68]	0.88	0.43	< 0.001
Lungs (mSv)	2.07 [0.72]	1.23 [0.14]	0.84	0.41	< 0.001
Skin (mSv)	0.61 [0.21]	0.39 [0.07]	0.22	0.36	< 0.001
Thyroid gland (mSv)	0.15 [0.09]	0.13 [0.08]	0.02	0.13	0.147
Red bone marrow (mSv)	0.65 [0.21]	0.42 [0.06]	0.20	0.31	< 0.001

Values are median [interquartile range], Difference = Value EID-CT − Value PCCT, Percentage difference = Difference / Value EID-CT

*CTDIvol* Volumetric CT-dose index, *DLP* Dose length product, *EID-CT* Energy-integrating detector CT, *PCCT* Photon-counting CT

## Discussion

This study assessed the performance of PCCT in comparison to a conventional EID-CT scanner in adult pwCF, focusing on radiation dose parameters as well as qualitative and quantitative aspects of image quality within an LD-HR chest CT scan protocol. The PCCT demonstrated an enhancement in qualitative image quality at approximately 42% lower radiation dose. In the following, the qualitative and quantitative image quality, the radiation dose, future research questions and the limitations of the study are discussed.

The following studies have qualitatively analysed the image quality of EID-CT and PCCT with different matrices. Bartlett et al investigated the influence of the 1,024 matrix between EID-CT and PCCT on the detection of small bronchi. In general, smaller bronchi were easier to detect on PCCT due to its better in-plane spatial resolution [28]. Jungblut et al compared the detection of pulmonary nodules on PCCT and EID-CT using an AI tool and concluded that the image quality of PCCT was superior [29]. Van Ballaer et al conducted a comparison of the image quality of PCCT and EID-CT for lung examinations using 512, 768, and 1,024 matrices. The study showed a consistent trend wherein images obtained with PCCT received higher ratings than those obtained with EID-CT. However, the study did not analyse the different matrices on the PCCT [30]. In summary, the findings of this study indicate an overall preference for PCCT images over EID-CT images. Additionally, the PCCT images received higher ratings with a 768 and 1,024 matrix compared to a 512 matrix. The similarity in ratings between the 768 and 1,024 matrices is likely due to the fact that the human eye cannot perceive the difference, and technical analysis tools are required to detect any variations. Furthermore, the anisotropy of voxels increases with a higher matrix, and thus the effect of better spatial in-plane resolution is limited by the slice thickness of 1 mm. Another approach conducted by Graafen et al and Milos et al on UHR chest CT is to select a slice thickness of 0.4 mm in order to achieve a better spatial in-plane resolution [31, 32].

Regarding quantitative image analysis, Woeltjen et al compared PCCT images of the chest with preliminary EID-CT examinations and determined the SNR. The SNR was found to be lower on PCCT than on EID-CT. However, the study did not analyse different matrices [22]. This study analysed the SNR between 512, 768, and 1,024 matrices. With our data, the SNR of the lung parenchyma was lower with PCCT than with EID-CT. Additionally, the 768 and 1,024 matrices had a significantly lower SNR than the 512 matrix. However, there was no significant difference between the 768 and 1,024 matrices. This may be because the small size of the patient population may have hindered the ability to obtain significant results for the minor difference. The results of Woeltjen et al and our results are in contrast to previous studies comparing the SNR of PCCT and EID-CT. Grunz et al analysed the SNR of bones and Decker et al of various abdominal organs. In both studies, PCCT SNR was significantly higher than EID-CT [33, 34]. One explanation for this may be the inhomogeneous lung parenchyma combined with the increased in-plane spatial resolution of PCCT, which can lead to increased noise [22]. The ROIs of the autochthonous back muscles were measured in a BI64 convolution kernel, therefore the noise is an order of magnitude higher than the signal.

Considering the relatively young age of this patient cohort and the significant lifetime radiation exposure they will accumulate due to the hereditary disease and the associated disease monitoring, it is important to consider the risk of radiation-induced malignancy. In this context, a recent study by Gomez et al showed a significantly increased risk of haematological malignancies in patients under 22 years of age with a single radiation exposure [35]. Studies of EID-CT have reported an effective radiation dose of 1.5–2.0 mSv for LD chest CT protocols [12, 36, 37]. In paediatric pwCF, Bayfield et al investigated the radiation dose for combined inspiratory and expiratory chest CT using LD and ultra-low-dose (ULD) protocols on EID-CT with a median of 0.66 mSv for the LD protocol and 0.15 mSv for the ULD protocol [23]. PCCT may offer further dose reduction in CT diagnostics in CF care. An average effective dose of 0.12 mSv has been shown in early studies of chest with PCCT in paediatric pwCF [38]. Initial studies of chest LD protocols using PCCT in adults found effective dose values of 1.4 mSv and a reduction in radiation dose of approximately 36% compared with EID-CT [22]. The dose values in this study were lower than previously published results for PCCT. The effective dose for adult pwCF in this study was 0.55 mSv, a reduction of 42% compared to EID-CT. This is particularly relevant given the increased risk of haematological malignancies in young patients. The effective dose for red bone marrow was 31% lower with PCCT than with EID-CT. The use of PCCT with an LD protocol can contribute to radiation protection in this patient population.

This study demonstrated that PCCT can achieve better image quality with a lower radiation dose than EID-CT using the same protocol parameters. Therefore, it may be relevant for future studies to further reduce the radiation dose while accepting some loss of image quality. When monitoring pwCF, it is important to assess infiltrates, bronchiectasis and bronchial wall thickening [8, 9, 11]. This can also be clinically sufficiently assessed with lower image quality in consultation with referring clinicians. For example, Suliman et al conducted a systematic literature review of radiation exposure for LD and ULD protocols in patients with COVID-19 pneumonia on EID-CT. The effective dose values for LD protocols ranged from 0.50 to 0.80 mSv, while for ULD protocols from 0.39 to 0.64 mSv on EID-CT [32]. Agostini et al investigated a ULD protocol with a pitch of 3 on EID-CT in chest CT of COVID-19 patients [33]. Greffier et al analysed an ULD protocol of 10 mAs on EID-CT in chest CT of patients with COVID-19 pneumonia [34]. Referring to our study on PCCT in pwCF with automatic tube current modulation and a pitch of 1.5, these are two aspects for further dose reduction for further research.

This study has some limitations. First, for an intraindividual comparison between the scanners, the number of pwCF was limited. Second, as longitudinal image data were analysed in this study due to radiation protection constraints, a head-to-head comparison with the latest EID-CT scanners was not possible. Here, especially a comparison of 1,024 matrix available in the the latest EID-CT scanners such as the SOMATOM Force (Siemens Healthineers, Erlangen, Germany) or from other manufacturers with advanced image reconstructions could yield interesting results [39]. Third, due to the retrospective nature of this study, the protocol based on EID-CT has already been established and transferred to PCCT. Phantom measurements would be useful to determine the optimal balance between adequate radiation dose and image quality.

In conclusion, LD-HR PCCT is an important technical innovation in the monitoring of pwCF, improving image quality by reducing radiation dose. Future studies may identify what parameters can be achieved with newer EID-CT and which benefits PCCT may have for malignancy risk in pwCF.

## Data Availability

The datasets used and analysed during the current study are available from the corresponding author upon reasonable request.

## Abbreviations

CF: Cystic fibrosis
CFTR: Cystic fibrosis transmembrane conductance regulator
CT: Computed tomography
CTDIvol: Volumetric CT-dose index
DLP: Dose length product
EID-CT: Energy-integrating detector system
IRR: Inter-rater reliability
LD: Low-dose
LD-HR: Low-dose high-resolution
PCCT: Photon-counting CT
pwCF: People with cystic fibrosis
ROI: Region of interest
SNR: Signal-to-noise ratio
ULD: Ultra-low-dose
